# Sustainable liquisolid compact system using fish viscera oil for enhanced dissolution and antioxidant activity of Baicalein

**DOI:** 10.1371/journal.pone.0358580

**Published:** 2026-09-21

**Authors:** Ruba Malkawi

**Affiliations:** Department of Pharmacy, Faculty of Pharmacy, Jadara University, Irbid, Jordan; Yantai Institute of Technology, CHINA

## Abstract

**Background:**

Baicalein (BA) is a flavonoid with significant pharmacological and antioxidant activities; however, its poor aqueous solubility and high crystallinity limit its dissolution and oral bioavailability. Liquisolid compact technology represents a promising approach for improving the dissolution behavior of poorly soluble drugs.

**Objective:**

This study aimed to develop a sustainable liquisolid compact formulation of BA using extracted fish viscera oil as a non-volatile solvent and to evaluate its physicochemical characteristics, dissolution behavior, and antioxidant activity.

**Methods:**

Fish oil was extracted from salmon viscera and used as a liquid vehicle for preparing BA liquisolid compacts using corn starch as a carrier and talc as a coating material. The prepared formulation was characterized using Fourier Transform Infrared Spectroscopy (FTIR), Differential Scanning Calorimetry (DSC), and Powder X-Ray Diffraction (PXRD) to evaluate drug–excipient interactions and changes in crystallinity. In vitro dissolution studies were conducted using USP apparatus II in phosphate buffer (pH 6.8). Antioxidant activity was assessed using the DPPH radical scavenging assay in a 96-well microplate reader.

**Results:**

FTIR analysis confirmed the absence of chemical incompatibilities between BA and the formulation components. DSC and PXRD analyses demonstrated a significant reduction in the crystalline nature of BA, indicating its transformation into an amorphous or molecularly dispersed state within the liquisolid compact. The liquisolid compact showed a marked improvement in drug dissolution compared with pure baicalein and the physical mixture. Furthermore, the fish oil–BA formulation exhibited enhanced antioxidant activity compared with BA alone.

**Conclusion:**

The developed fish oil–based liquisolid compact system significantly improved the dissolution and antioxidant performance of BA. In addition, the use of fish viscera oil provides a sustainable formulation approach by repurposing marine waste into a functional pharmaceutical excipient.

## 1. Introduction

The oral delivery of hydrophobic drugs remains one of the most significant challenges in pharmaceutical development, as low aqueous solubility often leads to poor bioavailability and inconsistent therapeutic outcomes [1].

Baicalein (5,6,7-trihydroxyflavone) is a flavonoid compound isolated from Scutellaria baicalensis (Chinese skullcap), a traditional Chinese medicinal herb that has been used for over 2,000 years to treat inflammatory conditions, fever, and infectious diseases [2, 3]. In traditional Chinese medicine, Scutellaria baicalensis root extracts have been valued for their therapeutic properties and incorporated into numerous herbal formulations [4]. In recent decades, modern pharmaceutical research has validated the traditional use of baicalein by confirming its diverse pharmacological properties. Baicalein exhibits potent anti-inflammatory activity through inhibition of nuclear factor-kappa B (NF-κB) signaling pathways, robust antioxidant activity via free radical scavenging and reactive oxygen species (ROS) neutralization, neuroprotective effects in neurodegenerative disease models, antimicrobial properties against pathogenic bacteria and viruses, and potential anticancer effects in various cancer cell lines [5]. These pharmacological properties make baicalein a promising candidate for the treatment of inflammatory diseases, neurodegenerative disorders, cancer, and infectious diseases.

Despite its therapeutic potential, baicalein has not been approved as a single-entity pharmaceutical drug by major regulatory agencies [6]. Instead, it is predominantly marketed as a bioactive constituent in botanical dietary supplements and herbal preparations, where product composition and declared content vary significantly among manufacturers [7]. This lack of standardized formulations has limited baicalein's clinical application and hindered the development of evidence-based therapeutic protocols [5]. However, the growing scientific evidence supporting baicalein's pharmacological activities has generated substantial commercial interest, with the global market for baicalein-containing products expanding rapidly, particularly in Asia and increasingly in Western markets [5]. This commercial demand, combined with scientific evidence for therapeutic efficacy, creates a compelling rationale for developing improved pharmaceutical formulations that can enhance baicalein's bioavailability and therapeutic performance.

The major barrier to the clinical development of baicalein is its poor aqueous solubility and high crystallinity, which severely limit its oral bioavailability [8,9]. Baicalein is classified as a Class II or III compound according to the Biopharmaceutics Classification System (BCS), indicating high permeability but low solubility [10]. The compound's lipophilicity (logP ≈ 2.5–3.0) indicates strong hydrophobicity and preferential partitioning into lipid phases rather than aqueous media. This poor water solubility results in minimal dissolution in the gastrointestinal tract, leading to incomplete absorption and high inter-individual variability in bioavailability [11]. These physicochemical limitations have restricted baicalein primarily to traditional herbal preparations with variable and often subtherapeutic baicalein content, preventing the realization of its full therapeutic potential.

The liquisolid compact technology represents a promising approach for enhancing the dissolution and bioavailability of poorly soluble drugs. This technique involves converting a liquid drug or drug solution into a dry, free-flowing powder by adsorption onto solid carriers and coating materials, thereby improving dissolution kinetics and converting crystalline drugs to amorphous or molecularly dispersed states. Fish oil, derived from marine sources, represents a particularly promising non-volatile vehicle for liquisolid formulations due to its natural origin, biocompatibility, and inherent bioactive properties rich in omega-3 polyunsaturated fatty acids (EPA and DHA) [12]. Furthermore, the use of fish viscera oil—a by-product of fish processing—as a pharmaceutical vehicle represents a sustainable and environmentally friendly approach by valorizing marine waste streams. The combination of baicalein with fish oil in a liquisolid formulation may therefore provide dual benefits: enhanced solubility and bioavailability of baicalein, coupled with the added therapeutic value of omega-3 fatty acids [13].

A key innovation and primary focus of this study is the integration of environmental sustainability and circular-economy principles into pharmaceutical manufacturing. While traditional liquisolid compacts often rely on synthetic non-volatile solvents, this research utilizes extracted fish viscera oil as a functional vehicle [14–16]. Fish viscera are typically considered a low-value waste product of the marine industry, yet they contain lipid components that can be “upcycled” into high-value pharmaceutical excipients [17]. By extracting this oil through controlled heating and centrifugation, we transform a discarded byproduct into a natural solubilizing agent designed to maintain BA in a molecularly dispersed state.

After successfully engineering these compacts, this research aims to prove that repurposed fish oil can significantly improve drug release compared to pure powder, while converting the drug from a crystalline to an amorphous state for superior absorption.

## 2. Materials and methods

### 2.1. Materials

Baicalein, Tween 80, Corn Starch, Talc, Magnesium Stearate and 2,2-diphenyl-1-picrylhydrazyl (DPPH) were all purchased from Sigma-Aldrich. The liquid vehicle was derived from fresh Salmon fish viscera, which were obtained from a local store. All other chemical reagents used were of analytical grade.

### 2.2. Extraction of salmon fish viscera oil

To promote sustainability and the reuse of marine waste products, a functional non-volatile vehicle was extracted from Salmon fish viscera. The fresh viscera were first minced into a uniform paste and then heated in a water bath at 70°C for 20 minutes to facilitate lipid separation. The mixture was subsequently centrifuged at 3000 rpm for 15 minutes. The resulting clear, yellow top layer of oil was collected and filtered through filter paper to remove any remaining solid impurities [12,13].

### 2.3. Pre-treatment of the carrier material

To optimize the oil-absorption capacity of the carrier, 50 g of commercial Corn Starch was spread on a metal tray and dried in a hot air oven at 80°C for 1 hour. This process removed intrinsic moisture, allowing the starch particles to function effectively as a “sponge.” The dried starch was then allowed to cool in a desiccator before use.

### 2.4. Formulation of Baicalein Liquisolid Compacts (10-Tablet Batch)

**Fig 1:**
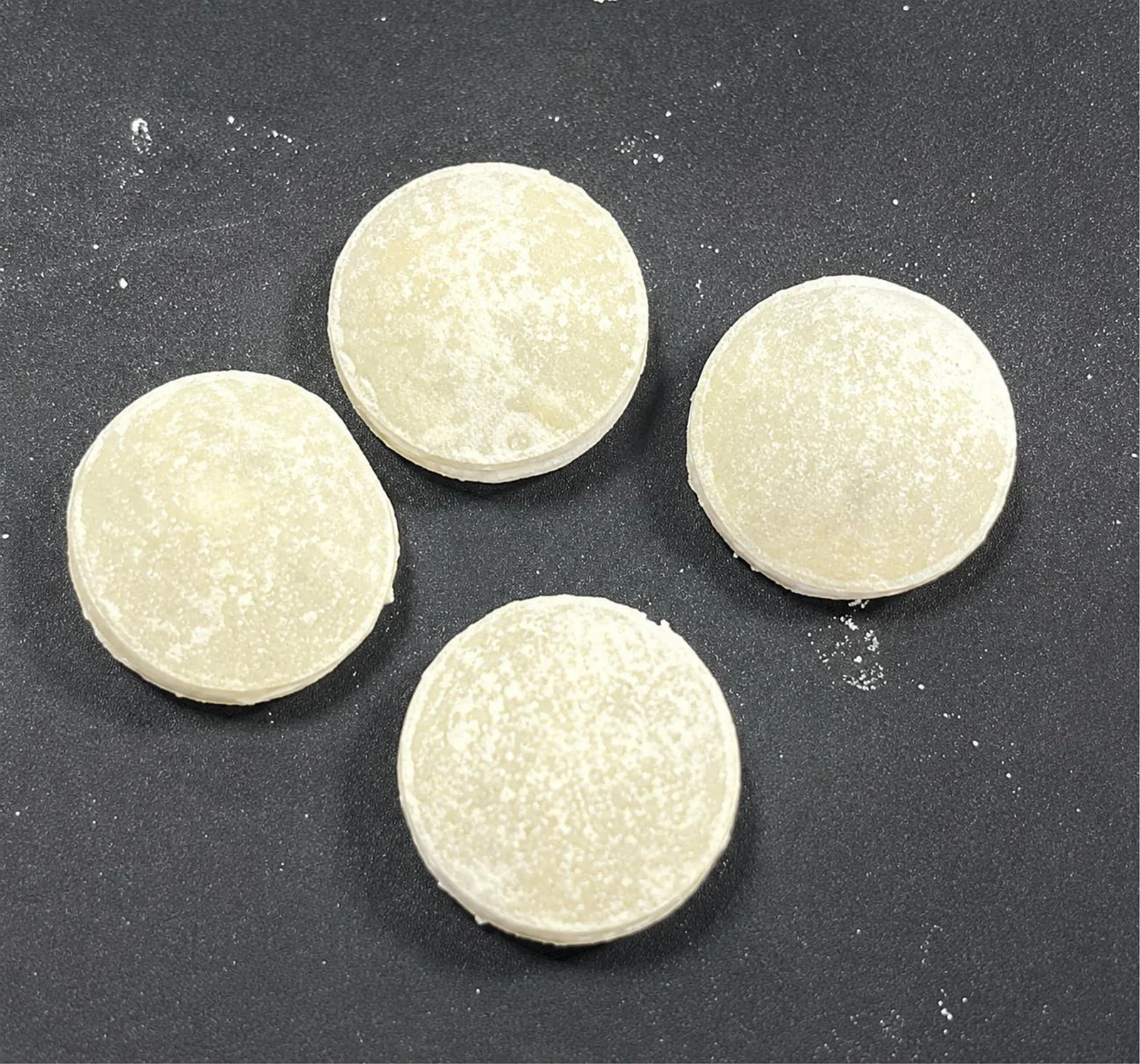
Sample of the compressed Liquidsolid compact disc.

The formulation was scaled down to prepare a pilot batch of 10 tablets due to material constraints (Fig 1).

The following specific weights were used to maintain the original ratio of ingredients (Fig 1):

- Step 1: Preparation of Liquid Medication: 0.5 g of BA was weighed and added to a mixture of 1.2 g of extracted Salmon fish oil and 0.3 g of Tween 80. The mixture was stirred continuously on a hot plate at 45–50°C for 10 minutes until a uniform oily suspension was formed.
- Step 2: Absorption Stage: 3.5 g of the pre-dried Corn Starch was placed in a porcelain mortar. The liquid medication was slowly added and mixed vigorously with a pestle for 5–10 minutes to ensure complete entrapment of the oil within the carrier.
- Step 3: Coating Stage: 0.75 g of Talc was added to the wet mass and triturated gently. This converted the damp mixture into a dry, free-flowing powder.
- Step 4: Final Blending: 0.2 g of extra (non-dried) Corn Starch was added as a disintegrant and mixed for 2 minutes. Finally, 0.05 g of Magnesium Stearate was added as a lubricant and mixed for exactly 30 seconds.
- Step 5: Compression: The resulting liquisolid powder was compressed into tablets using a single-punch machine equipped with a 12 mm or 13 mm die. The machine was adjusted to produce tablets with a target weight of approximately 650 mg [15].

**Fig 2:**
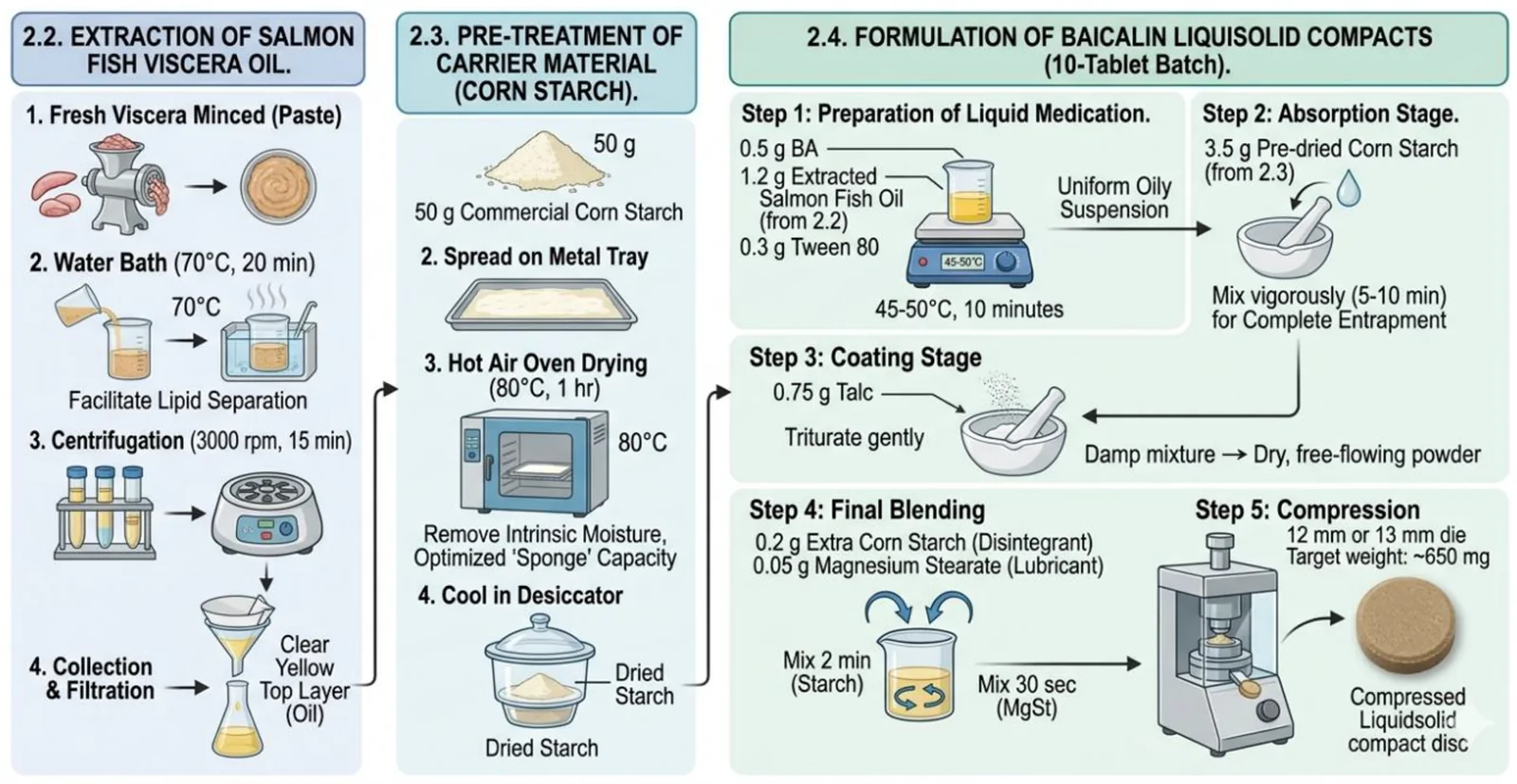
Graphical Scheme for the formulation of Baicalein liquisolid compacts from salmon viceral extract.

Fig 2 was generated using Google Gemini (https://gemini.google.com), a generative AI image generation tool. The image is published under the CC BY 4.0 license in accordance with Google's Terms of Service (https://policies.google.com/terms), which permit commercial use and publication of AI-generated content. No human-created artwork was modified or incorporated into this AI-generated figure.

### 2.5. Fourier Transform Infrared (FTIR) spectroscopy

FTIR analysis was performed to investigate potential intermolecular interactions and confirm the formation of complexes. FTIR spectra of pure BA, Talc, Corn Starch, Magnesium Stearate and the liquisolid compacts were recorded using an FTIR spectrophotometer (Shimadzu Corporation, Kyoto, Japan). For FTIR analysis, samples were ground to a fine powder and analysed using the KBr pellet method. Each spectrum was scanned in the range of 4000–400 cm^-1^ with a resolution of 4 cm^-1^and 32 scans. The resulting FTIR spectra were interpreted by comparing characteristic bands and identifying peak shifts, intensity changes, broadening, or disappearance of bands, which may indicate complex formation and intermolecular interactions [18].

### 2.6. Differential Scanning Calorimetry (DSC)

Thermal behaviour and physical state changes of FTIR spectra of pure BA, Talc, Corn Starch, Magnesium Stearate and the liquisolid compacts were evaluated using differential scanning calorimetry (DSC). Measurements were carried out using a Mettler Toledo DSC instrument (Mettler-Toledo International Inc., Greifensee, Switzerland). Accurately weighed samples (approximately 3–5 mg) were placed in standard aluminium pans and hermetically sealed, with an empty aluminium pan used as a reference. The samples were heated over a temperature range of 25–450°C at a heating rate of 10°C/min under a nitrogen purge atmosphere. Thermograms were analysed to identify changes in melting endotherms, peak shifts, or disappearance of characteristic peaks, which were considered evidence of successful complex formation and changes in crystallinity [18].

### 2.7. Powder X-Ray Diffraction (PXRD) analysis

Powder X-ray diffraction (PXRD) analysis was performed to investigate the crystalline characteristics and solid-state transformation of BA, physical mixtures, and the liquisolid compact. Diffraction patterns were recorded using an X-ray diffractometer equipped with Cu Kα radiation (λ = 1.5406 Å), operated at 40 kV and 30 mA. Samples were gently ground and uniformly spread on a sample holder to obtain a smooth surface. Data were collected over a 2θ range of 5°–70° with a step size of 0.02° and a scanning rate of 2°/min.

All measurements were conducted at room temperature. Diffractograms were analysed to compare peak positions, intensities, and crystallinity changes among pure BA, physical mixtures, and the prepared formula. Reduction in peak intensity, peak broadening, or disappearance of characteristic reflections was interpreted as evidence of reduced crystallinity or possible inclusion complex formation.

### 2.8. HPLC method

The quantification of BA was performed using a validated HPLC method as previously described by Malkawi et al. (submitted for publication) [19]. Chromatographic separation was achieved using a Diamonsil C18 column (150 mm × 4.6 mm, 5 μm) maintained at 40°C. The mobile phase consisted of 1% formic acid in water and acetonitrile (60:40, v/v), pumped at an isocratic flow rate of 1.0 mL/min. The injection volume was set at 35 μL, and UV detection was performed at 271nm.

The method was validated in accordance with ICH Q2(R1) guidelines. Linearity was established over a concentration range of 1–50 μg/mL with a correlation coefficient (R^2) of 0.9993. The method demonstrated high accuracy, with recovery rates ranging from 98.72% to 103.81% and a relative standard deviation (RSD) below 4%. Precision was confirmed with intra-day RSD values between 0.53% and 3.29% and inter-day RSD values between 0.23% and 1.60%.

### 2.9. *In Vitro* dissolution study

*In vitro* dissolution studies were conducted to evaluate the release behaviour of pure BA, physical mixture and the liquisolid compact. Dissolution testing was performed using a USP Apparatus II (paddle method). An accurately weighed amount of each formulation, equivalent to the same BA dose, was placed in 900 mL of dissolution medium maintained at 37 ± 0.5°C and stirred at a paddle rotation speed of 100 rpm. The dissolution medium consisted of phosphate buffer pH 6.8, selected to simulate intestinal conditions and ensure sink conditions [20,21]. Phosphate buffer pH 6.8 was selected as the dissolution medium to simulate intestinal conditions, where BA is primarily absorbed following oral administration. Since BA is a weakly acidic flavonoid with limited solubility in aqueous media, evaluation under near-neutral intestinal pH provides relevant insight into its dissolution-limited absorption behaviour. The use of 900 mL medium volume in USP Apparatus II ensured maintenance of sink conditions throughout the experiment and minimised saturation effects.

At predetermined time intervals (0, 10, 20, 30, 40, 50, and 60 min), 3 mL samples were withdrawn and immediately replaced with an equal volume of fresh, pre-warmed dissolution medium to maintain a constant volume. The withdrawn samples were filtered through a 0.45 µm membrane filter, suitably diluted if necessary, and analysed for BA content using HPLC at the predetermined λmax of 271 nm. The cumulative percentage of BA released was calculated and plotted as a function of time. Dissolution profiles of the different formulations were compared to assess the effect of the liquisolid in comparison with the BA alone [22,23]. Statistical analysis was performed using one-way analysis of variance (ANOVA) followed by Tukey’s post hoc test to compare dissolution performance between formulations at selected time points. Differences were considered statistically significant at p < 0.05. All experiments were conducted in triplicate, and data are presented as mean ± standard deviation (SD) [24,25].

### 2.10. Antioxidant

The antioxidant activity of the fish oil formulation containing BA and BA alone was evaluated using the 2,2-diphenyl-1-picrylhydrazyl (DPPH) radical scavenging assay using a 96-well microplate reader. A fresh DPPH solution was prepared by dissolving 2 mg of DPPH in 50 mL methanol to obtain a 0.1 mM solution, and the solution was protected from light using aluminum foil [26–28].

The fish oil–BA formulation and pure BA were dissolved in methanol containing 1% Tween-80 to enhance dispersion. Serial dilutions were prepared to obtain final concentrations of 25, 50, 100, 200, and 400 µg/mL.

For the assay, 100 µL of each sample solution was transferred into the wells of a 96-well microplate, followed by the addition of 100 µL of freshly prepared DPPH solution (0.1 mM). For the control, 100 µL of methanol was mixed with 100 µL of DPPH solution without sample. A blank containing methanol only was also prepared to correct the background absorbance.

The microplate was incubated in the dark at room temperature (25°C) for 30 min to allow the reaction between DPPH radicals and antioxidant compounds. After incubation, the absorbance was measured at 517 nm using a microplate reader. All experiments were performed in triplicate, and the mean values were used for analysis.

The percentage of DPPH radical scavenging activity was calculated using the following equation:


$$\begin{array}{c} \text{DPPH scavenging activity }(\%)=\frac{A_{control}-A_{sample}}{A_{control}}\times \text{1}\text{0}\text{0} \end{array}$$

where A control represents the absorbance of the control reaction and A sample represents the absorbance of the tested sample.

The IC₅₀ value, defined as the concentration required to inhibit 50% of the DPPH radicals, was calculated from the plot of percentage inhibition versus sample concentration.

## 3. Results

### 3.1. Fourier Transform Infrared (FTIR) spectroscopy

**Fig 3:**
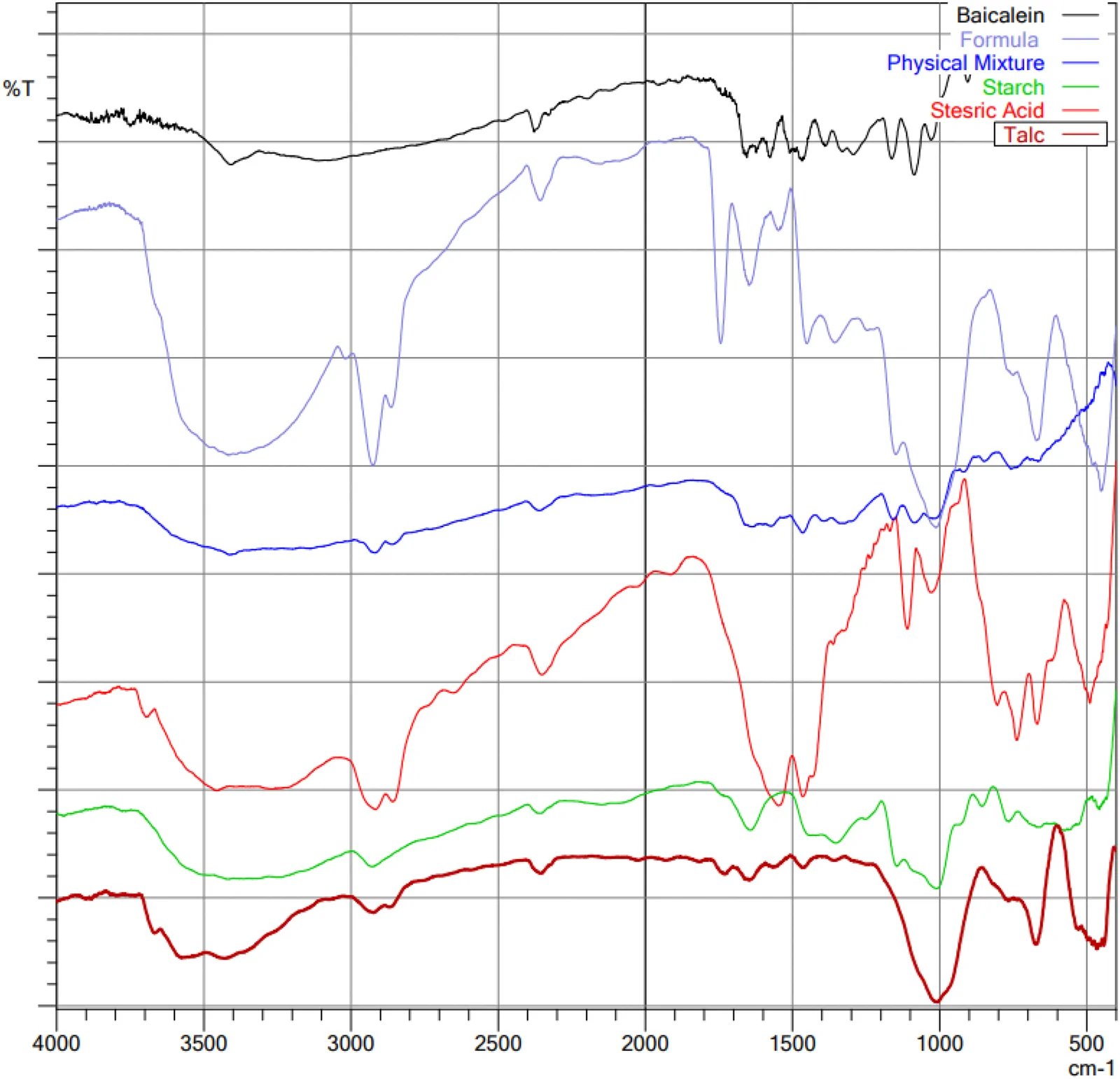
FTIR spectra for pure Baicalein, the Liquisolid compact, the physical mixture, Starch, Magnesium Stearate, and Talc.

FTIR analysis was conducted to evaluate the chemical compatibility between BA and the formulation excipients, including extracted fish oil, corn starch, and talc (Fig 3). The spectrum of pure BA displays its characteristic chemical “fingerprint” with distinct absorption bands. In the liquisolid compact, these characteristic peaks of BA remained visible, though some showed slight shifts in position or broadening. The absence of any new, unexpected peaks indicates that no chemical incompatibility or covalent reactions occurred between the drug and the fish oil vehicle [29].

### 3.2. Differential Scanning Calorimetry (DSC)

**Fig 4:**
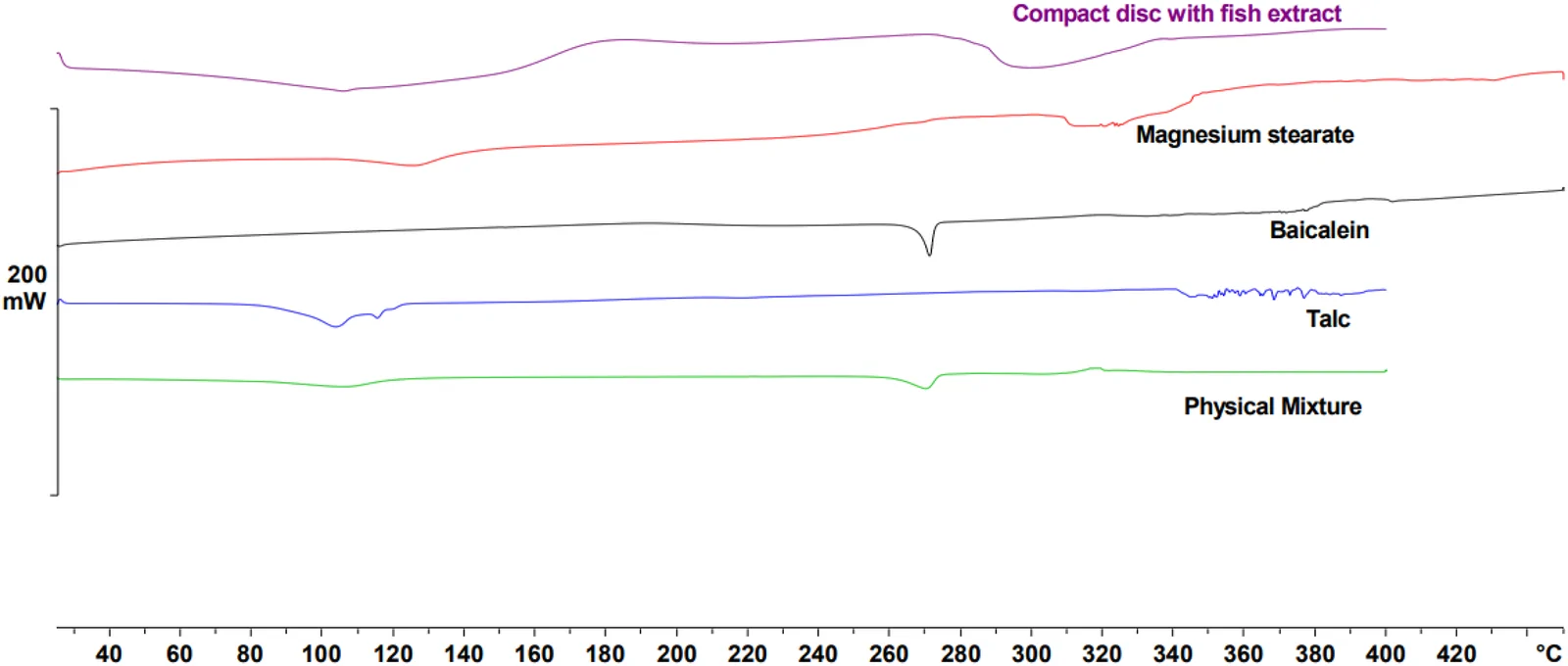
DSC thermograms of for pure Baicalein, the Liquisolid compact, the physical mixture, Starch, Magnesium Stearate, and Talc.

DSC thermograms were used to determine the physical state of the drug within the compacts. Pure BA exhibited a sharp endothermic melting peak, which is characteristic of its highly crystalline nature (Fig 4). This sharp peak was also observed in the physical mixture of the components. However, in the liquisolid compact formulation, this sharp melting peak significantly disappeared. The disappearance of the drug's crystalline peak indicates that BA was successfully converted from a crystalline to an amorphous or molecularly dispersed state within the fish oil matrix [30].

### 3.3. Powder X-Ray Diffraction (PXRD) analysis

**Fig 5:**
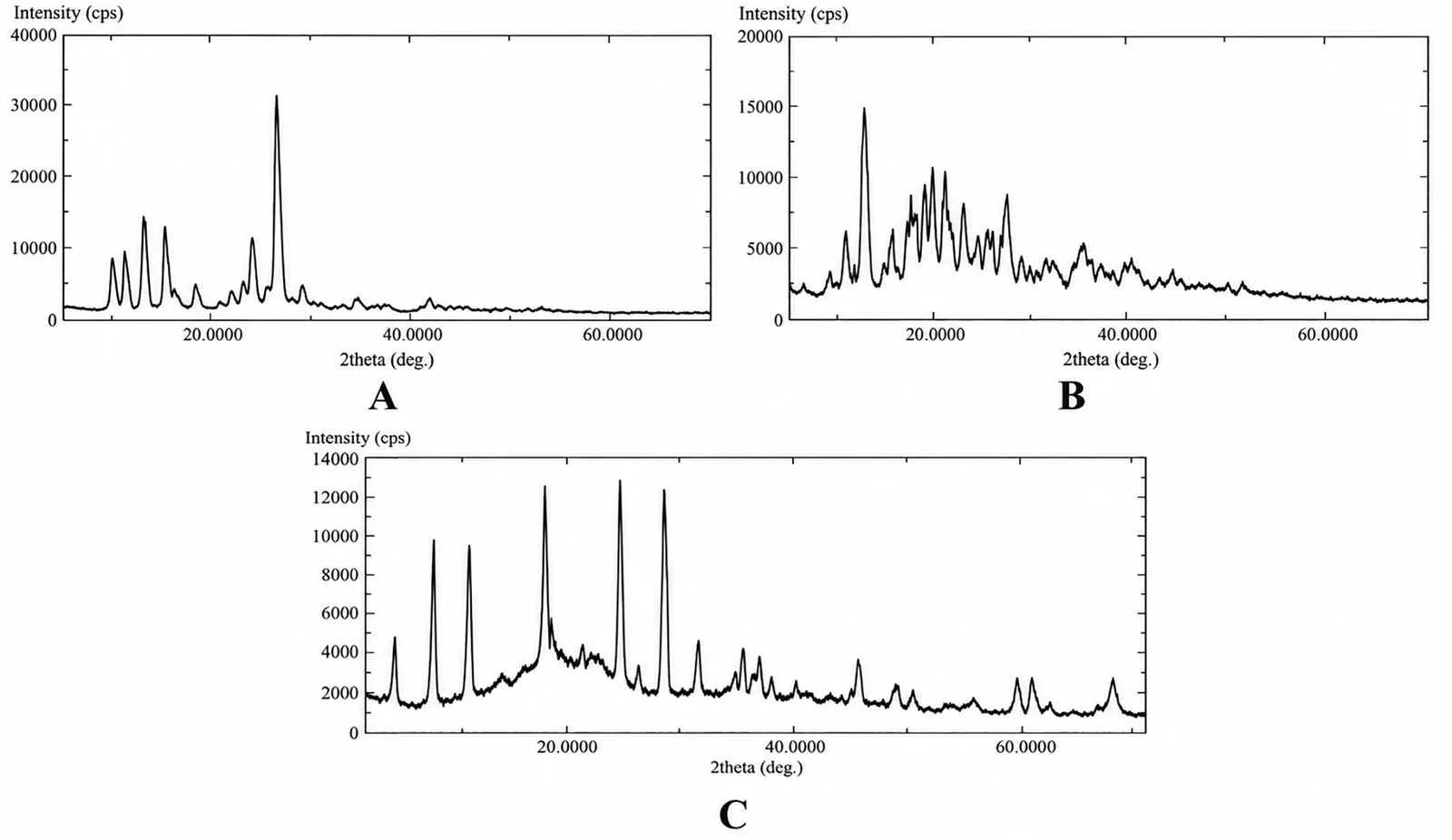
Powder X-ray diffraction (PXRD) patterns of (A) pure Baicalein and (B) physical mixture, and (C) Liquidsolid formula.

The PXRD patterns provided further evidence of the solid-state transformation. The diffractogram for pure BA showed high-intensity, sharp reflections, confirming its crystalline structure (Fig 5). While the physical mixture retained some of these crystalline peaks (at a lower intensity), the liquisolid compact (Fish oil formulg) exhibited a significant reduction in peak intensity and noticeable peak broadening. This reduction in crystallinity supports the DSC findings, proving the drug exists primarily in a non-crystalline state within the formulation [31,32].

### 3.4. *In Vitro* Dissolution Study

**Fig 6:**
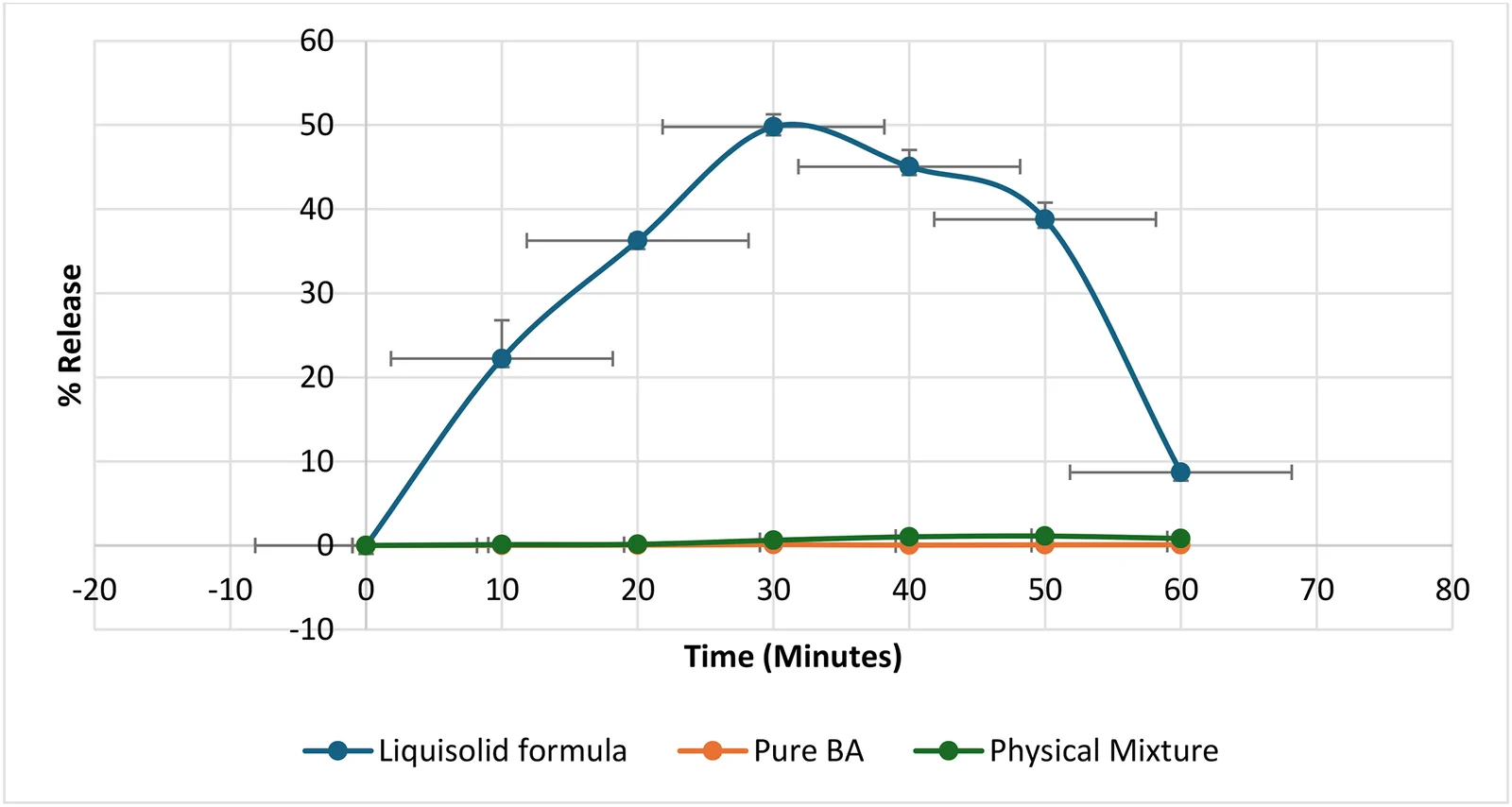
In vitro dissolution profiles of pure BA, the liquisolid compact and the physical mixture in phosphate buffer pH 6.8, Error bars representing mean ± SD (n = 3). All the values.

The *in vitro* dissolution profiles in phosphate buffer (pH 6.8) demonstrated significantly enhanced drug release for the liquisolid compact compared to pure baicalein and the physical mixture (Fig 6). The fish oil-based liquisolid formulation achieved a maximum cumulative drug release of 49.8% at 30 minutes, representing a 450-fold enhancement compared to pure baicalein (0.11% at 30 min) and an 80-fold improvement over the physical mixture (0.62% at 30 min).

The liquisolid formulation displayed rapid initial dissolution, with 22.2% drug release within 10 minutes, increasing to 36.3% at 20 minutes and reaching peak dissolution at 30 minutes. In contrast, pure baicalein showed negligible release (0.01% at 10 min, 0.04% at 20 min, 0.11% at 30 min), while the physical mixture demonstrated only minimal improvement (0.10% at 10 min, 0.14% at 20 min, 0.62% at 30 min).

The dissolution profile of the liquisolid compact showed a peak at 30 minutes followed by a decrease at later time points, reflecting the approach to equilibrium in the dissolution medium as the system becomes saturated. This pattern is characteristic of poorly soluble drugs and confirms successful solubilization of baicalein by the fish oil vehicle. The superior dissolution performance of the liquisolid formulation compared to the physical mixture demonstrates that the fish oil vehicle provides molecular dispersion of baicalein rather than simple physical mixing, as confirmed by DSC and PXRD analyses. These results indicate that the liquisolid technology effectively overcomes the poor aqueous solubility of baicalein and provides a promising platform for enhanced oral bioavailability [33].

### 3.5. Antioxidant activity

The antioxidant activity of fish oil, BA, and the fish oil–BA liquisolid compact was evaluated using the DPPH radical scavenging assay in a 96-well microplate system (Table 1). The absorbance of the DPPH control solution was recorded at 0.28 ± 0.007, indicating the initial radical concentration in the reaction mixture [34,35].

**Table 1:** DPPH radical scavenging activity of fish oil, baicalein, and fish oil–BA liquisolid compact after 30 min incubation. Data are presented as mean ± SD (n = 3).

Sample	Absorbance (517 nm) Mean ± SD (n = 3)	% Inhibition
Control (DPPH)	0.28 ± 0.007	—
Fish Oil (blank)	0.202 ± 0.003	27.86
BA	0.160 ± 0.015	42.86
Fish Oil–BA Formula	0.108 ± 0.005	61.43

The blank fish oil sample showed moderate antioxidant activity, with an average absorbance of 0.202 ± 0.003, corresponding to approximately 27.86% DPPH inhibition. This effect may be attributed to the presence of natural lipid components in fish oil, including omega-3 fatty acids and minor bioactive compounds that possess mild antioxidant properties.

BA alone exhibited stronger radical scavenging activity with an absorbance value of 0.160 ± 0.015, corresponding to 42.86% inhibition of DPPH radicals. This observation is consistent with the known antioxidant activity of BA, which contains multiple phenolic hydroxyl groups capable of donating hydrogen atoms to neutralize free radicals.

Interestingly, the fish oil–BA liquisolid compact demonstrated the highest antioxidant activity, with an absorbance value of 0.108 ± 0.005 corresponding to 61.43% inhibition of DPPH radicals. The enhanced radical scavenging activity may be attributed to improved dispersion and solubilization of BA within the lipid matrix, which facilitates greater interaction between the antioxidant molecules and DPPH radicals.

These findings indicate that incorporation of BA into the fish oil-based liquisolid system enhances its antioxidant performance, likely due to improved molecular dispersion and the combined antioxidant contributions of both the flavonoid and lipid components.

## 4. Discussion

The primary challenge associated with the oral delivery of BA lies in its poor aqueous solubility and high crystallinity, which significantly limit its dissolution rate and consequently its bioavailability [36,37]. In this study, a liquisolid compact system utilizing repurposed fish viscera oil as a non-volatile solvent was successfully developed to overcome these limitations. The formulation strategy aimed to maintain BA in a molecularly dispersed state within a lipid matrix while simultaneously converting a low-value marine by-product into a functional pharmaceutical excipient [38].

The spectroscopic and thermal analyses confirmed the successful incorporation of BA into the liquisolid system without chemical degradation. FTIR analysis demonstrated that the characteristic peaks of BA remained present in the formulation, indicating the absence of chemical interactions between the drug and the excipients [12]. The slight shifts and peak broadening observed in the spectra suggest physical interactions and hydrogen bonding within the formulation matrix rather than chemical incompatibility. These findings confirm that the formulation process preserved the structural integrity of BA while enabling its dispersion within the fish oil vehicle [4,5].

Thermal analysis using DSC provided strong evidence of a significant change in the physical state of BA in the liquisolid compact. Pure BA exhibited a sharp melting endothermic peak characteristic of its crystalline structure. In contrast, the disappearance of this peak in the liquisolid compactindicates a transformation of the drug from a crystalline to an amorphous or molecularly dispersed state. This transformation is critical because amorphous drug forms generally possess higher free energy and greater apparent solubility compared with their crystalline counterparts [39,40].

The PXRD results further supported the DSC findings. The diffractogram of pure BA displayed sharp, intense peaks typical of a highly crystalline material. In contrast, the liquisolid compact showed a marked reduction in peak intensity and broadening of diffraction peaks, confirming a substantial reduction in crystallinity [10]. This reduction indicates that the drug molecules were successfully dispersed within the lipid matrix, resulting in a more disordered structure. The conversion of BA from a crystalline to a less ordered state plays a fundamental role in enhancing dissolution performance.

The in vitro dissolution results clearly demonstrated the effectiveness of the liquisolid formulation in improving the release profile of BA. While pure BA exhibited extremely poor dissolution due to its crystalline nature and low solubility, the liquisolid compacts showed a substantially faster and higher drug release [41,42]. The improved dissolution behavior can be attributed to several factors inherent to the liquisolid technique. First, the drug is maintained in a solubilized state within the fish oil vehicle, eliminating the need for the dissolution medium to penetrate and break down a crystalline lattice. Second, the high surface area provided by the carrier material facilitates rapid exposure of the drug to the dissolution medium. Finally, the presence of Tween 80 improves wettability and dispersion, further enhancing drug release.

The use of dried corn starch as a carrier played a critical role in the successful formation of the liquisolid system. The porous structure of the starch particles allowed efficient absorption of the liquid medication, effectively converting the oily drug dispersion into a dry and free-flowing powder suitable for compression. The addition of talc as a coating material further improved flowability by reducing interparticle friction, enabling the preparation of tablets with acceptable mechanical properties.

In addition to improving dissolution, the antioxidant evaluation provided further insight into the functional properties of the developed formulation. BA alone exhibited significant DPPH radical scavenging activity due to its polyphenolic structure and the presence of hydroxyl groups capable of donating hydrogen atoms to neutralize free radicals. The blank fish oil also showed moderate antioxidant activity, which can be attributed to the presence of bioactive lipid components such as omega-3 fatty acids and naturally occurring antioxidant compounds [43].

Interestingly, the fish oil–BA formulation demonstrated the highest antioxidant activity among the tested samples. This enhanced radical scavenging effect can be explained by the improved molecular dispersion of BA within the lipid matrix, which increases its availability to interact with free radicals in the reaction medium. Additionally, the lipid environment may facilitate better distribution of the antioxidant molecules and contribute to a synergistic antioxidant effect between BA and the lipid components of fish oil.

From a formulation perspective, the improved antioxidant activity is consistent with the dissolution findings and further supports the hypothesis that molecular dispersion of BA within the fish oil vehicle enhances its physicochemical and functional properties. By maintaining the drug in a more readily available form, the liquisolid system allows greater interaction with both dissolution media and reactive radical species.

Beyond the pharmaceutical advantages, the use of fish viscera oil as a formulation vehicle represents an important contribution to sustainable pharmaceutical development. Fish processing waste is often discarded despite containing valuable lipid components. By extracting and repurposing this oil as a drug delivery vehicle, the present study demonstrates an innovative strategy aligned with circular economy principles. This approach not only improves drug performance but also promotes environmental sustainability through the valorization of marine by-products [44].

The fish oil-based liquisolid technology demonstrated in this study for baicalein has significant potential applicability to a broad range of poorly soluble pharmaceutical compounds. Approximately 40% of newly developed drugs exhibit poor aqueous solubility, representing a major challenge in pharmaceutical development. The liquisolid approach is particularly well-suited for Biopharmaceutics Classification System (BCS) Class II and Class III compounds (high permeability, low solubility), which constitute a substantial portion of the pharmaceutical pipeline. Other poorly soluble flavonoids, such as quercetin, kaempferol, and apigenin, share similar physicochemical properties to baicalein (high lipophilicity, poor aqueous solubility, crystalline nature) and would likely benefit from the fish oil-based liquisolid formulation approach. Additionally, non-flavonoid hydrophobic drugs, including certain terpenoids, steroids, and lipophilic xenobiotics, could potentially be formulated using this technology. The successful development of a fish oil-based liquisolid system for baicalein therefore provides a proof-of-concept that can guide the development of similar formulations for other poorly soluble compounds.

While the fish oil-based liquisolid technology demonstrates broad applicability, certain criteria should be considered when selecting candidate drugs for formulation using this approach. Ideal candidates would include: (1) compounds with logP values in the range of 2–5, indicating sufficient lipophilicity for solubilization in fish oil; (2) drugs with molecular weights less than 500 Da, facilitating molecular dispersion within the lipid matrix; (3) compounds that are chemically stable in the presence of fish oil and the selected carriers and coating materials; (4) drugs with therapeutic doses less than 500 mg, as larger doses may exceed the solubilization capacity of the liquisolid system; and (5) compounds that do not undergo significant first-pass metabolism or that benefit from enhanced absorption. Drugs that meet these criteria and are currently formulated as poorly bioavailable conventional dosage forms represent priority candidates for liquisolid reformulation. Examples include curcumin, resveratrol, and ginkgo biloba flavonoids.

Beyond the immediate application to baicalein formulation, this study has several broader implications for pharmaceutical science and drug development. First, it demonstrates the potential of sustainable, nature-derived materials (fish oil from marine waste) as pharmaceutical excipients, contributing to the growing movement toward green pharmacy and sustainable pharmaceutical manufacturing. The use of fish viscera oil—a by-product of fish processing—represents an innovative approach to waste valorization and circular economy principles in pharmaceutical development.

Second, the combination of baicalein with omega-3 rich fish oil creates a synergistic therapeutic formulation that may offer enhanced therapeutic benefits compared to baicalein alone. The anti-inflammatory and antioxidant properties of EPA and DHA in fish oil complement and potentially enhance baicalein's pharmacological effects, particularly for inflammatory and oxidative stress-related diseases. This multi-component approach to drug formulation, where the excipient vehicle itself possesses therapeutic activity, represents an innovative paradigm that could be applied to other drug-excipient combinations.

Third, this study contributes to the growing body of evidence supporting the use of liquisolid technology for poorly soluble natural products and phytopharmaceuticals. Many traditional herbal medicines contain poorly soluble bioactive compounds, and the development of effective formulation strategies for these compounds could facilitate the transition from traditional herbal preparations to standardized, bioavailable pharmaceutical products. This could enhance the therapeutic efficacy of traditional medicines and support their integration into evidence-based medical practice.

Fourth, the successful development of this formulation has implications for the pharmaceutical industry's approach to drug development. Rather than focusing exclusively on chemical synthesis of novel compounds, the pharmaceutical industry increasingly recognizes the value of optimizing the formulation of existing bioactive compounds to enhance their therapeutic utility. This “formulation innovation” approach can extend the patent life of compounds, improve clinical outcomes, and reduce development costs compared to de novo drug discovery.

To extend the findings of this study and validate the broader applicability of the fish oil-based liquisolid technology, several future research directions are recommended. First, in vivo pharmacokinetic studies in appropriate animal models should be conducted to determine whether the enhanced in vitro dissolution translates into improved bioavailability and therapeutic efficacy. Second, the formulation should be optimized through systematic variation of oil-to-carrier ratios, carrier types, and coating materials to identify the optimal formulation composition. Third, comprehensive stability studies should be conducted under various storage conditions to establish shelf-life and storage requirements. Fourth, the applicability of the fish oil-based liquisolid approach should be evaluated for other poorly soluble flavonoids and drug classes to confirm its broad utility. Finally, the therapeutic efficacy of the liquisolid formulation should be evaluated in appropriate disease models to determine whether the enhanced bioavailability translates into improved clinical outcomes.

## 5. Conclusion

This study successfully developed a fish oil–based liquisolid compact system for BA aimed at improving its dissolution behavior and functional performance. The use of extracted fish viscera oil as a non-volatile vehicle allowed BA to be maintained in a molecularly dispersed and partially amorphous state, as confirmed by DSC and PXRD analyses. FTIR results further demonstrated the absence of chemical incompatibilities between the drug and formulation excipients.

The liquisolid compact significantly enhanced the in vitro dissolution profile of BA compared with the pure drug and the physical mixture, indicating that the liquisolid technique effectively overcomes the dissolution limitations associated with the crystalline form of the drug. In addition, the antioxidant evaluation showed that the fish oil–BA formulation exhibited superior radical scavenging activity compared with BA alone, suggesting improved functional performance of the formulation.

Furthermore, the use of fish viscera oil as a pharmaceutical vehicle represents a sustainable and environmentally friendly approach by transforming a marine by-product into a valuable formulation component. Overall, the results demonstrate that fish oil-based liquisolid systems represent a promising strategy for enhancing the solubility, dissolution, and antioxidant potential of poorly water-soluble flavonoids such as BA, providing a sustainable platform for future pharmaceutical applications.

## 6. Study limitations

This study has several limitations that should be acknowledged. First, the evaluation was limited to in vitro characterization, including FTIR, DSC, PXRD, dissolution testing, and antioxidant assessment, and no in vivo bioavailability study was performed. Therefore, although the developed fish oil-based liquisolid system showed improved dissolution and antioxidant performance, these findings cannot yet confirm enhanced oral absorption or therapeutic performance in vivo. Second, long-term stability studies were not conducted, and thus the physical and chemical stability of the formulation during storage remains to be established. Future studies should include pharmacokinetic evaluation and accelerated as well as long-term stability testing to confirm the reliability and practical applicability of the developed formulation.

## Supporting information

S1 Fig Graphical Abstract. (DOCX)

S1 Appendix A Fig 6. Dissolution values the formula, pure drug and physical mixture. Word data set containing the concentration values for the dissolution with their standard deviation in triplicate used to draw Fig 6.
(DOCX)

## References

[1] Singh N, Singh AP, Singh AP. Solubility: An Overview. International Journal of Pharmaceutical Chemistry and Analysis. 2021;7:166–71.

[2] Bajek-Bil A, Chmiel M, Włoch A, Stompor-Gorący M. Baicalin-Current Trends in Detection Methods and Health-Promoting Properties. Pharmaceuticals (Basel). 2023;16(4):570. doi: 10.3390/ph16040570 37111327 PMC10146343

[3] Ozma AM, Khodadadi E, Farzaneh P, Kamounah SF, Yousefi M. Baicalin, a natural antimicrobial and anti-biofilm agent. J Herb Med. 2021;27:1–15.

[4] Yan T, Ji M, Sun Y, Yan T, Zhao J, Zhang H, et al. Preparation and characterization of baicalein/hydroxypropyl-β-cyclodextrin inclusion complex for enhancement of solubility, antioxidant activity and antibacterial activity using supercritical antisolvent technology. J Incl Phenom Macrocycl Chem. 2019;96(3–4):285–95. doi: 10.1007/s10847-019-00970-2

[5] Liu H. The fascinating effects of baicalein on cancer: A review. Int J Mol Sci. 2016;17:1–17. 10.3390/ijms17101681 PMC5085714 27735841

[6] Shi S. Chemical characterization of extracts of leaves of Kadsua coccinea (Lem.) A.C. Sm. by UHPLC-Q-Exactive Orbitrap Mass spectrometry and assessment of their antioxidant and anti-inflammatory activities. Biomedicine & Pharmacotherapy. 2022;149:112828. 10.1016/j.biopha.2022.112828 35339830

[7] Tan X, Chen P, Xiao L, Gong Z, Qin X, Nie J, et al. Extraction, purification, structural characterization, and anti-inflammatory activity of a polysaccharide from Lespedeza formosa. Int J Biol Macromol. 2025;300:140154. doi: 10.1016/j.ijbiomac.2025.140154 39855506

[8] Sameena Y, Chandrasekaran S, Israel V M V E. Inclusion complexation between baicalein and β-cyclodextrin and the influence of β-cyclodextrin on the binding of baicalein with DNA: a spectroscopic approach. J Biomol Struct Dyn. 2016;34(7):1395–408. doi: 10.1080/07391102.2015.1082148 26308145

[9] Gharari Z, Bagheri K, Danafar H, Sharafi A. Simultaneous determination of baicalein, chrysin and wogonin in four Iranian Scutellaria species by high performance liquid chromatography. Journal of Applied Research on Medicinal and Aromatic Plants. 2020;16:100232. doi: 10.1016/j.jarmap.2019.100232

[10] Paczkowska-Walendowska M, Rył A, Kwiatek J, Rosiak N, Szarzyński K, Wawrzyniak W, et al. Baicalein-Loaded Chitosan Films for Local Treatment of Oral Infections. Polymers (Basel). 2025;17(16):2167. doi: 10.3390/polym17162167 40871115 PMC12389395

[11] Malkawi R, Jarwan B, Alhawarri MB, Isawi IH, Banisalman K, Alghodran O, et al. β-Cyclodextrin-chitosan based carriers enhance the solubility and dissolution of baicalein through inclusion complex formation. Sci Rep. 2026;16(1):23877. doi: 10.1038/s41598-026-56841-7 42270847 PMC13434384

[12] Zhang Y, Sun Q, Liu S, Wei S, Xia Q, Ji H, et al. Extraction of fish oil from fish heads using ultra-high pressure pre-treatment prior to enzymatic hydrolysis. Innovative Food Science & Emerging Technologies. 2021;70:102670. doi: 10.1016/j.ifset.2021.102670

[13] Khoshnoudi-Nia S, Forghani Z, Jafari SM. A systematic review and meta-analysis of fish oil encapsulation within different micro/nanocarriers. Crit Rev Food Sci Nutr. 2022;62(8):2061–82. doi: 10.1080/10408398.2020.1848793 33207958

[14] Spireas S. Liquisolid systems and methods of preparing same. 1996.

[15] Javadzadeh Y, Musaalrezaei L, Nokhodchi A. Liquisolid technique as a new approach to sustain propranolol hydrochloride release from tablet matrices. Int J Pharm. 2008;362(1–2):102–8. doi: 10.1016/j.ijpharm.2008.06.022 18647643

[16] Javadzadeh Y, Jafari-Navimipour B, Nokhodchi A. Liquisolid technique for dissolution rate enhancement of a high dose water-insoluble drug (carbamazepine). Int J Pharm. 2007;341(1–2):26–34. doi: 10.1016/j.ijpharm.2007.03.034 17498898

[17] Estiasih T, Ahmadi K, Ali D, Nisa F, Suseno S, Lestari L. Valorisation of viscera from fish processing for food industry utilizations. IOP Conf Ser: Earth Environ Sci. 2021;924(1):012024. doi: 10.1088/1755-1315/924/1/012024

[18] Nakhla DS, Naguib YW, Saha S, Gao D, Gupta N, Malkawi W, et al. Cyclodextrin-Based Inclusion Complexes Improve the In Vitro Solubility and Pharmacokinetics of Ivacaftor Following Oral Administration in Mice. AAPS PharmSciTech. 2025;26(5):135. doi: 10.1208/s12249-025-03131-6 40379997 PMC12765454

[19] Malkawi R. Development and validation of a robust HPLC method for quantification of baicalin. BMC Chemistry. 2026.

[20] Malkawi R. Evaluating artificial intelligence tools in the pharmaceutical industry: A case study on paracetamol dissolution and calibration curves. Res J Pharm Technol. 2025;18:2269–74.

[21] Malkawi R, Al-Olimat S, Tawalbeh J. Unlocking the potential: Enhancing solubility and bioavailability of acyclovir through solid dispersion formulations. Int J App Pharm. 2024;:111–8. doi: 10.22159/ijap.2024v16i5.51313

[22] Malkawi R, Battah K, Alkhreisat M. Pharmaceutical Insights Into Ammi and Parsley: Evaluating Antioxidant Activity, Total Phenolic Content, and Kidney Stone Disintegration Properties. Adv Pharmacol Pharm Sci. 2025;2025:5522905. doi: 10.1155/adpp/5522905 40018327 PMC11867721

[23] Jarwan B, Tawalbeh J, Malkawi R. Assessment of Phenol and Antioxidant Content of Olive Varieties and Their Potential Health Benefits for Colon Health. ScientificWorldJournal. 2023;2023:9165902. doi: 10.1155/2023/9165902 37868295 PMC10586902

[24] Hussein LS, Al-Khedairy EBH. Solubility and Dissolution Enhancement of Ebastine by Surface Solid Dispersion Technique. IJPS. 2021;30(1):122–32. doi: 10.31351/vol30iss1pp122-132

[25] Khadka P, Ro J, Kim H, Kim I, Kim JT, Kim H, et al. Pharmaceutical particle technologies: An approach to improve drug solubility, dissolution and bioavailability. Asian Journal of Pharmaceutical Sciences. 2014;9(6):304–16. doi: 10.1016/j.ajps.2014.05.005

[26] Maqsood S, Benjakul S, Abushelaibi A, Alam A. Phenolic Compounds and Plant Phenolic Extracts as Natural Antioxidants in Prevention of Lipid Oxidation in Seafood: A Detailed Review. Comp Rev Food Sci Food Safe. 2014;13(6):1125–40. doi: 10.1111/1541-4337.12106

[27] Nafiu MO, Salawu MO, Kazeem MI. Antioxidant Activity of African Medicinal Plants. Medicinal Plant Research in Africa. Elsevier. 2013. p. 787–803. doi: 10.1016/b978-0-12-405927-6.00021-7

[28] Gigliobianco MR, Cortese M, Nannini S, Di Nicolantonio L, Peregrina DV, Lupidi G, et al. Chemical, Antioxidant, and Antimicrobial Properties of the Peel and Male Flower By-Products of Four Varieties of Punica granatum L. Cultivated in the Marche Region for Their Use in Cosmetic Products. Antioxidants (Basel). 2022;11(4):768. doi: 10.3390/antiox11040768 35453453 PMC9030693

[29] Eid MM. Characterization of Nanoparticles by FTIR and FTIR-Microscopy. Handbook of Consumer Nanoproducts. Springer Singapore. 2021. p. 1–30. doi: 10.1007/978-981-15-6453-6_89-1

[30] Lin S-Y. Current and potential applications of simultaneous DSC-FTIR microspectroscopy for pharmaceutical analysis. J Food Drug Anal. 2021;29(2):182–202. doi: 10.38212/2224-6614.3345 35696204 PMC9261823

[31] Mathers A, Pechar M, Hassouna F, Fulem M. The step-wise dissolution method: An efficient DSC-based protocol for verification of predicted API-polymer compatibility. Int J Pharm. 2023;648:123604. doi: 10.1016/j.ijpharm.2023.123604 37981251

[32] Yang H, Wierzbicki M, Du Bois DR, Nowick JS. X-ray Crystallographic Structure of a Teixobactin Derivative Reveals Amyloid-like Assembly. J Am Chem Soc. 2018;140(43):14028–32. doi: 10.1021/jacs.8b07709 30296063 PMC6356018

[33] Baboota S, Dhaliwal M, Kohli K. Physicochemical characterization, in vitro dissolution behavior, and pharmacodynamic studies of rofecoxib-cyclodextrin inclusion compounds. preparation and properties of rofecoxib hydroxypropyl beta-cyclodextrin inclusion complex: a technical note. AAPS PharmSciTech. 2005;6(1):E83–90. doi: 10.1208/pt060114 16353967 PMC2750415

[34] Malkawi R, Jarwan B, Waleed R, Younis R. Pharmaceutical prospects of pomegranate antioxidants in combating microbial infections. International Journal of Food Properties. 2024;27(1):768–82. doi: 10.1080/10942912.2024.2360985

[35] Singleton VL, Orthofer R, Lamuela-Raventós RM. [14] Analysis of total phenols and other oxidation substrates and antioxidants by means of folin-ciocalteu reagent. Methods in Enzymology. Elsevier. 1999. p. 152–78. doi: 10.1016/s0076-6879(99)99017-1

[36] Shaji J, Patole V. Protein and Peptide drug delivery: oral approaches. Indian J Pharm Sci. 2008;70(3):269–77. doi: 10.4103/0250-474X.42967 20046732 PMC2792531

[37] Zhang L, Wang S, Zhang M, Sun J. Nanocarriers for oral drug delivery. J Drug Target. 2013;21(6):515–27. doi: 10.3109/1061186X.2013.789033 23621127

[38] Liquisolid systems and methods of preparing same. 1996.

[39] Bhujbal SV, Mitra B, Jain U, Gong Y, Agrawal A, Karki S, et al. Pharmaceutical amorphous solid dispersion: A review of manufacturing strategies. Acta Pharm Sin B. 2021;11(8):2505–36. doi: 10.1016/j.apsb.2021.05.014 34522596 PMC8424289

[40] Lopez FL, Shearman GC, Gaisford S, Williams GR. Amorphous formulations of indomethacin and griseofulvin prepared by electrospinning. Mol Pharm. 2014;11(12):4327–38. doi: 10.1021/mp500391y 25317777

[41] Amidon GL, Lennernäs H, Shah VP, Crison JR. A theoretical basis for a biopharmaceutic drug classification: the correlation of in vitro drug product dissolution and in vivo bioavailability. Pharm Res. 1995;12(3):413–20. doi: 10.1023/a:1016212804288 7617530

[42] Baokar S, Khapake K, Sonavane S, Pawar V, Rode M. New dissolution method for the evaluation of acyclovir using pH 7.4 phosphate buffer in-vitro and determination of its content by validated UV spectrophotometric method. Research J Pharma Dosage Forms and Tech. 2013;5:47–9.

[43] Al-Zharani M, Rudayni H, Mubarak M, Alkahtani S, Nasr F, Albatli S, et al. Omega-3 fatty acids (EPA/DHA) act as a natural antioxidant to alleviate cadmium-induced oxidative stress and restore the oxidation/antioxidant balance in male Wistar mice. Ital J Food Sci. 2026;38(1):270–85. doi: 10.15586/ijfs.v38i1.3243

[44] Reddy KB, Chintagunta AD, Gutti RK, Sampath Kumar NS. Sustainable valorization of fish viscera into omega-3 rich lipids and their functional validation. AMB Express. 2026;16(1):35. doi: 10.1186/s13568-026-02029-1 41721148 PMC13031517

