# Supplementary material for: Sustainable liquisolid compact system using fish viscera oil for enhanced dissolution and antioxidant activity of Baicalein
Source: PLoS One. 2026 Sep 21;21(9):e0358580. doi: 10.1371/journal.pone.0358580 (PMC13592643; doi:10.1371/journal.pone.0358580)
Supplement: Supplementary file 1 [file pone.0358580.s001.docx]

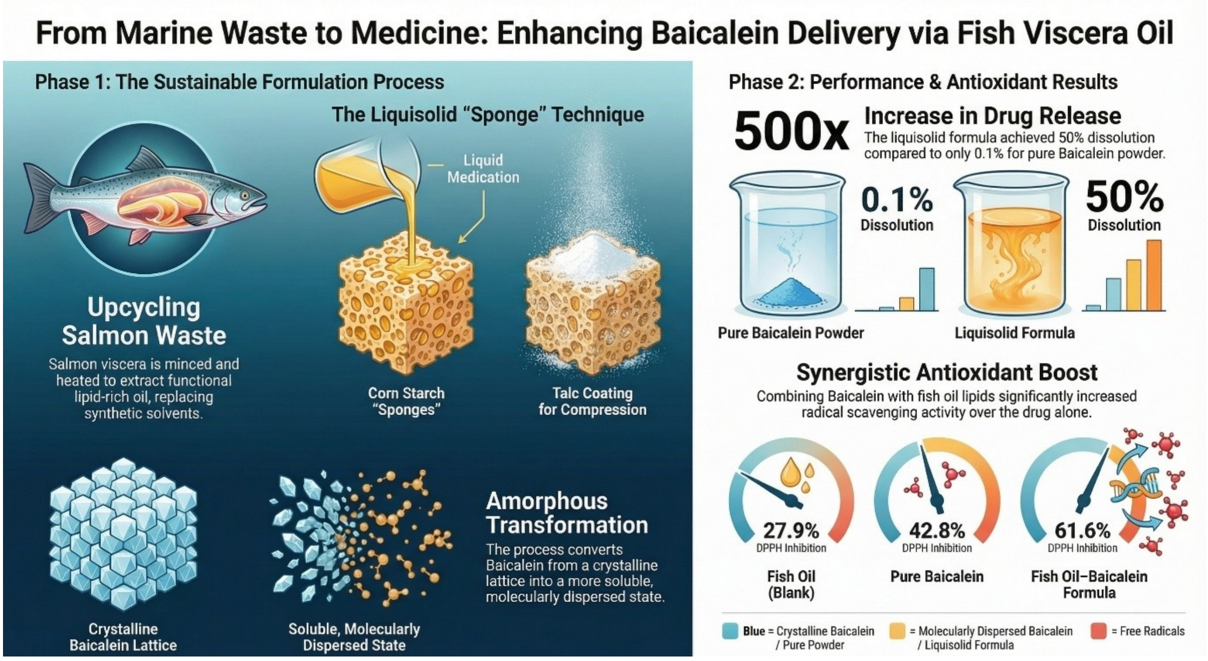
From Marine Waste to Medicine: Enhancing Baicalein Delivery via Fish Viscera Oil
Phase 1: The Sustainable Formulation Process
The Liquisolid "Sponge" Technique
Liquid Medication
Upcycling Salmon Waste
Salmon viscera is minced and heated to extract functional lipid-rich oil, replacing synthetic solvents.
Corn Starch "Sponges"
Talc Coating for Compression
Amorphous Transformation
The process converts Baicalein from a crystalline lattice into a more soluble, molecularly dispersed state.
Crystalline Baicalein Lattice
Soluble, Molecularly Dispersed State
Phase 2: Performance & Antioxidant Results
500x
Increase in Drug Release
The liquisolid formula achieved 50% dissolution compared to only 0.1% for pure Baicalein powder.
0.1% Dissolution
50% Dissolution
Pure Baicalein Powder
Liquisolid Formula
Synergistic Antioxidant Boost
Combining Baicalein with fish oil lipids significantly increased radical scavenging activity over the drug alone.
27.9% DPPH Inhibition
Fish Oil (Blank)
42.8% DPPH Inhibition
Pure Baicalein
61.6% DPPH Inhibition
Fish Oil–Baicalein Formula
Blue = Crystalline Baicalein / Pure Powder
= Molecularly Dispersed Baicalein / Liquisolid Formula
= Free Radicals
