# Supplementary material for: Sustainable liquisolid compact system using fish viscera oil for enhanced dissolution and antioxidant activity of Baicalein
Source: PLoS One. 2026 Sep 21;21(9):e0358580. doi: 10.1371/journal.pone.0358580 (PMC13592643; doi:10.1371/journal.pone.0358580)
Supplement: Supplementary file 2 [file pone.0358580.s002.docx]

**S1 Appendix A**

**Dissolution values for Fig 6**

Table 1: Dissolution values for Liquisolid formula (n=3).

| Time (Minutes) | Liquisolid formula (Concentration trail 1) (µg/ml) | Liquisolid formula (Concentration trail 2) (µg/ml) | Liquisolid formula (Concentration trail 3) (µg/ml) | Average Concentration (µg/ml) | Standard deviation |
| --- | --- | --- | --- | --- | --- |
| 0 | 0 | 0 | 0 | 0 | 0 |
| 10 | 7.080970564 | 14.59773294 | 15.34947683 | 12.34272678 | 4.572290299 |
| 20 | 20.34480141 | 20.79580715 | 19.28794462 | 20.14285106 | 0.773951105 |
| 30 | 29.15102679 | 27.64774948 | 26.17897411 | 27.65925013 | 1.486059716 |
| 40 | 23.52900719 | 27.28212664 | 24.27602303 | 25.02905229 | 1.986646976 |
| 50 | 22.30067801 | 19.29393548 | 23.05242942 | 21.5490143 | 1.988798111 |
| 60 | 4.674802309 | 5.367088608 | 4.465077122 | 4.835656013 | 0.472029253 |

Table 2: Dissolution values for physical mixture formula (n=3).

| Time (Minutes) | Liquisolid formula (Concentration trail 1) (µg/ml) | Liquisolid formula (Concentration trail 2) (µg/ml) | Liquisolid formula (Concentration trail 3) (µg/ml) | Average Concentration (µg/ml) | Standard deviation |
| --- | --- | --- | --- | --- | --- |
| 0 | 0 | 0 | 0 | 0 | 0 |
| 10 | 0.000105235 | 0.082789621 | 0.089637391 | 0.057510749 | 0.016921557 |
| 20 | 0.097657777 | 0.059998797 | 0.08295499 | 0.080203855 | 0.012535283 |
| 30 | 0.296949698 | 0.38038576 | 0.361067681 | 0.34613438 | 0.017172404 |
| 40 | 0.379776902 | 0.515266544 | 0.812103491 | 0.569048979 | 0.158156156 |
| 50 | 0.764341983 | 0.557029676 | 0.540665684 | 0.620679114 | 0.042271307 |
| 60 | 0.313268589 | 0.680793169 | 0.389894465 | 0.461318741 | 0.151598432 |

Table 2: Dissolution values for the pure baicalein (n=3).

| Time (Minutes) | Liquisolid formula (Concentration trail 1) (µg/ml) | Liquisolid formula (Concentration trail 2) (µg/ml) | Liquisolid formula (Concentration trail 3) (µg/ml) | Average Concentration (µg/ml) | Standard deviation |
| --- | --- | --- | --- | --- | --- |
| 0 | 0 | 0 | 0 | 0 | 0 |
| 10 | 4.51006E-05 | 0.004277038 | 0.011884001 | 0.005402047 | 0.005999094 |
| 20 | 0.003630596 | 0.028586247 | 0.035185965 | 0.022467603 | 0.016643728 |
| 30 | 0.001142548 | 0.082706936 | 0.095312547 | 0.059720677 | 0.051120185 |
| 40 | 0.006043477 | 0.035802339 | 0.027428666 | 0.023091494 | 0.015346197 |
| 50 | 0.020159957 | 0.005983343 | 0.102836826 | 0.042993375 | 0.052308442 |
| 60 | 0.00605851 | 0.035937641 | 0.110353589 | 0.050783247 | 0.053709029 |
