# Supplementary material for: Sustainable liquisolid compact system using fish viscera oil for enhanced dissolution and antioxidant activity of Baicalein
Source: PLoS One. 2026 Sep 21;21(9):e0358580. doi: 10.1371/journal.pone.0358580 (PMC13592643; doi:10.1371/journal.pone.0358580)
Supplement: Supplementary file 3 [file pone.0358580.s004.docx]

Thanks for the comment;

**Comment:**

1. We note that Figure 2 is created using a generative AI software. Please provide a direct link to the Terms and Conditions of the AI software to confirm that generated images can be published under CC BY 4.0 license.

Contributions by artificial intelligence (AI) tools and technologies to a study or to an article’s contents must be clearly reported in the legend of the corresponding figure or supplementary file. This section should include the name(s) of any tools used, noting which panels are created using the generative AI and any attributions as dictated by the Terms of Use of the software.

**Response:**

New legend was added to Figure 2

The legend:

Figure 2 was generated using Google Gemini (<https://gemini.google.com>), a generative AI image generation tool. The image is published under the CC BY 4.0 license in accordance with Google's Terms of Service (<https://policies.google.com/terms>), which permit commercial use and publication of AI-generated content. No human-created artwork was modified or incorporated into this
